# FBXO2 targets glycosylated SUN2 for ubiquitination and degradation to promote ovarian cancer development

**DOI:** 10.1038/s41419-022-04892-9

**Published:** 2022-05-07

**Authors:** Jing Ji, Jing Shen, Yuxin Xu, Mengru Xie, Qilan Qian, Teng Qiu, Wen Shi, Dexu Ren, Jinming Ma, Wei Liu, Bin Liu

**Affiliations:** 1 Jiangsu Key Laboratory of Marine Pharmaceutical Compound Screening, College of Pharmacy, Jiangsu Ocean University, Lianyungang, 222005 China; 2 Department of Obstetrics and Gynecology, Jingzhou Hospital Affiliated to Yangtze University, Jingzhou, Hubei China

**Keywords:** Ovarian cancer, Ubiquitin ligases

## Abstract

SAD1/UNC84 domain protein-2 (SUN2) plays a tumor suppressor role in various types of cancer by inhibiting cancer cell proliferation, migration and promoting apoptosis. However, the post-translational regulation of SUN2 and the cellular mechanism responsible for its proteasomal degradation remains largely unknown. Here, we show that FBXO2, an E3 ubiquitin ligase of the F-box proteins (FBPs) family targets glycosylated SUN2 for ubiquitination and degradation via the ubiquitin-proteasome system (UPS). By integrating the Cancer Genome Atlas (TCGA), Gene Expression Omnibus (GEO), and the Encyclopedia of Cancer Cell Lines (CCLE) databases, we revealed that FBXO2 was selectively highly expressed in ovarian cancer (OV) tissues and cells. Patients with relatively high FBXO2 expression levels were associated with worse prognosis. Manipulation of the expression of FBXO2 affecting ovarian cancer cell proliferation, migration/invasion in vitro, and tumor growth in mice in vivo. The transcription factor SOX6 promoted FBXO2 expression by recognizing a putative response element localized on the promoter region of FBXO2. Abnormally highly expressed FBXO2 recognized and targeted glycosylated SUN2 protein for ubiquitination-depended degradation to prevent cell apoptosis, promote cell proliferation, and ultimately promote the progression of OV. Thus, we revealed a new SOX6-FBXO2-SUN2 axis that contributed to the development of OV, and targeting this axis may represent an effective OV treatment strategy.

## Introduction

The ubiquitin-proteasome system (UPS) is one of the main cellular systems that degrade and remove redundant proteins to maintain protein homeostasis [1]. The selectivity of UPS is determined by the E3 ligases. Among them, the cullin–RING ligases (CRLs) constitute the largest class E3 ligases with more than 200 documented members [2]. The CRL1 ligases, also known as the SKP1–Cullin 1–F-box protein (SCF) complexes, are the best characterized among all CRLs.

The aberrant expression or mutation of F-box proteins will lead to the abnormal accumulation or degradation of its substrate proteins that often involved in tumorigenesis [3, 4]. Previously, we have reported that F-box family members FBXO6 [5] and FBXO16 [6] act as oncogenes or tumor suppressor genes in ovarian cancer (OV), respectively. Here, we show that FBXO2 is a new player involved in the process of OV tumorigenesis by targeting glycosylated SUN2 for destruction via UPS.

## Materials and methods

### Data acquisition

In this study, the GEPIA database (http://gepia.cancer-pku.cn/) was used to analyze the differential expression of F-box proteins in ovarian cancer (a *p* < 0.01 & log2|fold change |>1 was set as the cutoff values), the expression of FBXO2 in ovarian cancer tissues and normal tissues. Three microarray datasets GSE66957 (including 57 ovarian cancer tissue samples and 12 non-cancerous ovarian tissue samples), GSE69428 (including 10 ovarian cancer tissue samples and 10 non-cancerous ovarian tissue samples), and GSE32062 (including 260 ovarian cancer tissue samples) were downloaded from GEO and from which the mRNA expressions of FBXO2 were analyzed by R software. The CCLE database was used to analyze the expression of FBXO2 mRNA in ovarian cancer cell lines. The online software Kaplan–Meier Plotter (http://kmplot.com/) was used to analyze the relationship between FBXO2 gene expression and overall survival (OS), progression-free survival (PFS), and post-progression survival (PPS) of patients with ovarian cancer.

### Cell culture

HEK293T cells, ovarian cancer cell lines A2780 cells and OVCAR8 cells were purchased from American Type Culture Collection with and tested for mycoplasma contamination. All cells were cultured in high-glycemic Dulbecco’s modified Eagle’s medium (DMEM) or 1640 (Invitrogen) containing 10% fetal bovine serum (FBS), 100 units/mL penicillin, and 100 mg/mL streptomycin. The culture conditions were set at 37 °C and 5% CO_2_.

### RNA interference, RNA isolation, and real-time PCR

The lentiviral human shRNA plasmids against SOX6 or FBXO2 were purchased from Sigma-Aldrich (https://www.sigmaaldrich.cn/). The lentiviral particles were used to infected ovarian cancer cells and cells stably expressing shRNAs were selected by puromycin for about two weeks. The target sequences of the plasmids were as follows: SOX6-shRNA1: CCAACACTTGTCAGTACCATT; SOX6-shRNA2: GCCACACATTAAGCGACCAAT; FBXO2-shRNA1: CACCGTTAAGCTACTGTCCGA; FBXO2-shRNA2: TCGTGGTGAAGGACTGGTACT. Total RNA of A2780 cells was extracted with Trizol (Invitrogen, USA) lysis reagent, followed by cDNA synthesis using a reverse transcription kit. RT-PCR was performed using a Light Cycler 480 (Roche, Switzerland). The relative expression of target genes was analyzed by the 2-ΔΔCt method using β-actin as an internal reference gene. FBXO2 forward, 5′- GTGTCGCAAAGCACAGGTC-3′; reverse, 5′-CGGACAGTAGCTTAACGGTGAG-3′; SOX6 forward, 5′-GGATGCAATGACCCAGGATTT-3′; reverse, 5′- TGAATGGTACTGACAAGTGTTGG-3′; SUN2 forward, 5′- TGACGTGCCTGACGTATGG -3′; reverse, 5′- AAATGTGGCGATGAGTCTCTG-3 ′; β-actin forward, 5′-CATGTACGTTGCTATCCAGGC-3′; reverse, 5′-CTCCTTAATGTCACGCACGAT-3′.

### sgRNAs and CRISPR-CAS9 assay

We used the CRISPR/Cas9 technology to generate knockout cells by using the design from the CCTOP website (https://crispr.cos.uni-heidelberg.de/) as previously described [7]. The sgRNA score more than 0.70 was chosen. FBXO2: forward primer: CACCG-AACCTTCTGCGTAACCCGTG, reverse primer: AAACCACGGGTTACGCAGAAGGTTC. SUN2: forward primer: CACCGAGTCGCTGGTCCACGAGTCC, reverse primer: AAACGGACTCGTGGACCAGCGACTC. Oligonucleotides were annealed and cloned into PX459 vector. Cells were transfected with indicated sgRNA plasmid by Lipofectamine 2000. After 48 h, cells were transferred into 96-well plates to obtain individual clones. The genomic fragments containing the target nucleotide sequence in the center were PCR-amplified and sequenced. The protein depletion was identified by immunoblotting.

### Western blotting

Total proteins were collected after cells were lysed with the SDS lysis buffer (100 mM Tris-HCl, pH 6.8, 100 mM DTT, 1% SDS, 10% glycerol), and the protein concentration were assayed. The proteins were separated by electrophoresis using 10–12% SDS-PAGE, transferred to NC membranes, and blocked in TBS containing 5% skim milk at room temperature for 1 h. The membranes were washed three times with TBST for 5 min each time and then incubated with primary antibodies at 4 °C overnight. After washing the membrane with TBST, the secondary antibody was added and incubated for 1 h. After washing with TBST, chemiluminescence development was performed. The following primary antibodies were used: anti-FBXO2 (A-12, sc-393873, Santa Cruz), anti-Sox-6 (A-4, sc-393314, Santa Cruz), anti-SUN2 (G-5, sc-377459, Santa Cruz), anti-GST (B-14, sc-138, Santa Cruz), anti-FLAG (m2, Sigma-Aldrich), anti-HA (Sigma-Aldrich), and anti-β-actin (C-2, sc-8432, Santa Cruz).

### Immunohistochemical (IHC) staining

Ten paraffin-embedded specimens of human ovarian and cancer tissues were obtained from Jingzhou Hospital of Yangtze University and with appropriate patient consent. IHC staining of the paraffin-embedded normal and tumor tissues was performed using anti-FBXO2 and anti-SUN2 primary antibodies, respectively. An ABC Elite immunoperoxidase kit was used according to the manufacturer’s instructions. Double-blind readings were performed by two pathologists, and staining intensity were scored.

### Immunoprecipitation (IP) and GST-pulldown

Cells transfected with the appropriate plasmids were collected and lysed in IP buffer (100 mM NaCl, 20 mM Tris-cl PH8.0, 0.5 mM EDTA, 0.5% (v/v) Nonidet P-40) on ice for 20 min and then sonicated for 20 s. Lysates were cleared by centrifugation at 14,000 rpm for 10 min, then the supernatant was incubated with the appropriate antibody overnight, followed by incubation with Protein A and G Sepharose (Beyotime, China) for 2 h at 4 °C. The beads were washed three times with IP buffer, denatured at 95 °C for 5 min, and then separated on 12% SDS-PAGE for immunoblot analysis. For GST-pulldown assay, HA-SUN2 protein was produced in HEK293T cells transiently transfected with HA-SUN2 plasmids and then immunoprecipitated with anti-HA resin, eluted with 150 ng/ml of HA peptide (Sigma-Aldrich). GST or GST tagged FBXO2 proteins were incubated with HA-SUN2 in PB buffer (20 mM HEPES, pH 7.5, 130 mM KCl, 5 mM MgCl2, 1 mM DTT, 0.5 mM EDTA, 0.05% NP40) and rotated at 30 °C for 2 h. Glutathione beads were then added and incubated for another 30 min. The beads were then washed with PB buffer, treated with SDS buffer, boiled for 5 min, and separated by SDS-PAGE for immunoblotting analysis.

### Plasmids, Luciferase, and ChIP assay

The FBXO2 WT and FBXO2 MUT plasmids have been described previously [8]. The SOX6 and SUN2 genes were cloned into expression vectors by using cDNAs from 293 T or A2780 cells. The promoter region of FBXO2 gene was amplified from the genomic DNA of A2780 cells and cloned into the pGL4.15 vector (Promega, Madison, Wisconsin, USA). For transcriptional regulation of FBXO2 by SOX6, cells were transiently transfected with pGL4.15-FBXO2 WT or MUT and pGL4.15-Renilla with or without SOX6. Cells were harvested, lysate was added, and luciferase activity was assayed using the Dual Luciferase Assay System (Promega, Madison, WI). The firefly luciferase luminescence data were normalized by the Renilla luciferase luminescence data. For Chip assay, we used a ChIP assay kit (Upstate, Billerica, MA) as previously described [9]. Cells were fixed with formaldehyde and DNA was sheared to fragments at 100–500 bp by sonication. The supernatants were then incubated and precipitated with antibodies against SOX6 or normal serum IgG overnight and rotationally incubation at 4 °C. The eluted product was purified with a DNA purification kit to obtain purified DNA, which was used as the template to perform PCR. The GPADH promoter was used as negative control.

### Colony formation assay

Cells were inoculated in six-well plates at 1000 per well and cultured until colony formation was visible. The colonies were fixed with paraformaldehyde for 15–30 min and stained with crystal violet for 30 min. The number of colonies was recorded and the colony formation rate was calculated. The number of colonies of control cells was set to 1. Data are shown as the mean ± SD of more than three independent experiments.

### Cell proliferation assays

2 × 10^4^ cells were inoculated in 96-well cell culture plates, and 10 μL CCK⁃8 reagent was added at 24, 48, and 72 h, respectively, and cultured at 37 °C. The absorbance values were measured at 450 nm wavelength and the relative cell viability was calculated.

### Cell migration and invasion assay

For the migration assay, A2780 cells were inoculated in six-well plates for 24 h. When the cells reached ~80% confluence, the bottom of each well was scribed with a 10 μl gun tip, then washed three times with PBS to remove floating cells, and 2 mL of serum-free medium was added to each well. Photographs were taken under the microscope at the same position at 0, 24, and 48 h after scratching, and the migration area was calculated using Image J software. For the invasion assay, 50 mg/mL of Matrigel 1:8 dilution was applied to the upper surface of the bottom membrane of the Transwell and air-dried at 4 °C. After the cells were collected, the cell concentration was adjusted to 1 × 10^5^ cells/mL with serum-free DMEM medium. 200 μL of cell suspension was added to the Transwell and 500 μL of DMEM medium containing 10% FBS was added to the lower chamber of the 24-well plate, and the cells were incubated for 24 h. After wiping off the non-migrating cells, the cells were stained with crystal violet for 20 min and observed under an inverted microscope. The number of invading cells was determined by taking the average value of five randomly selected fields of view for counting and photographing.

### In vivo ubiquitination assay

The Tandem Ubiquitin Binding Entity (K48 TUBE HF (GST)) based in vivo ubiquitination assay has been described elsewhere [5–7]. Briefly, cells after treatment were lysed with IP buffer (100 mM NaCl, 20 mM Tris-cl PH8.0, 0.5 mM EDTA, 0.5% (v/v) Nonidet P-40) with protease and phosphorylate inhibitors for 30 min on ice. Cells were sonicated and the lysates were centrifuged. The supernatant was incubated with K48 TUBE HF (GST) (LifeSensors) for 2 h at 4 °C in a rotating wheel and glutathione beads were then added and incubated for 1 h. Then, the beads were then washed with IP buffer and boiled. Boiled samples were separated by SDS-PAGE and subjected to immunoblotting with indicated antibodies.

### Flow cytometry

Each group of cells was collected. The cell concentration was adjusted to 1 × 10^5^ cells/mL using appropriate binding buffer. 100 μL of cell suspension was incubated with 5 μL of Annexin-V-FITC and 5 μL of PI for 30 min at room temperature in the dark. 500 μL of cell suspension was replenished, and the cell apoptosis was detected by flow cytometry within 1 h.

### Xenograft assays

The animal study was approved by the Ethics Committee of Jiangsu Ocean University. The BALB/c nude mice were purchased from National Rodent Laboratory Animal Resources (Shanghai, China) and housed at specific facility. Mice were randomly divided into two groups, with 6 mice in each group. Suspensions of A2780 or SKOV3 cells (1 × 10^7^ cells) with or without FBXO2 silencing were prepared with 50 µl DMEM medium, and injected subcutaneously into the corresponding group of nude mice. The tumor volume was calculated by the formula length × width 2 × 1/2 weekly. The mice were sacrificed four weeks later when the biggest tumors grew to about 1000 mm^3^, and the tumors were removed to detect the tumor weight, and finally photographed.

### Statistical analysis

GraphPad Prism 8.0 statistical software was used. Quantitative data were expressed as mean ± standard deviation and were normally distributed. T-test for independent samples was used for comparison between two groups, and one-way ANOVA was used for comparison between multiple groups. The differences were considered statistically significant at * *P* < 0.05, ** *P* < 0.01 and *** *P* < 0.001.

## Results

### Upregulation of FBXO2 correlates with poor prognosis for patients with OV

**Fig. 1:**
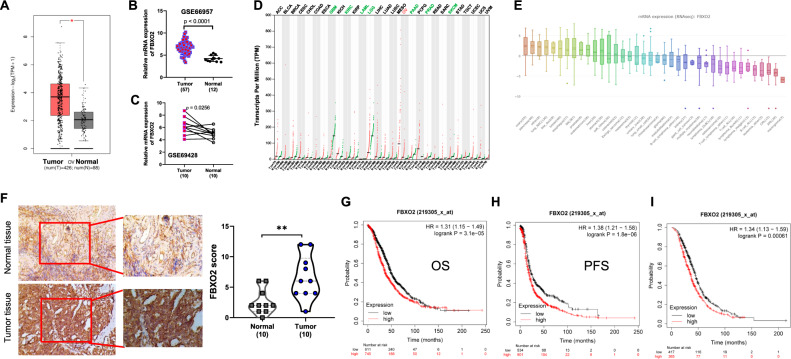
Upregulation of FBXO2 correlates with poor prognosis for OV patients. **A** The mRNA expression of FBXO2 in TCGA OV and GTEx datasets through the GEPIA website (http://gepia2.cancer-pku.cn/). Red represents OV tissue and black represents normal tissue. **B** Relative mRNA expression of FBXO2 in GSE66957 dataset. Red represents OV tissue and black represents normal tissue. **C** Relative mRNA expression of FBXO2 in GSE66428 dataset. **D** Dot plot showed FBXO2 gene expression profile in 29 tumor types and matched normal tissues from TCGA and GTEx datasets. Each point represents the expression of the sample. **E** The mRNA expression of FBXO2 in cell lines from different cancer types obtained through the CCLE website (https://sites.broadinstitute.org/ccle). **F** The representative IHC staining images (scale bars, 100 μm) and the expression scores of FBXO2 in normal ovarian tissues and OV tissues from 10 OV patients. **G**, **H**. The overall survival (OS) (**G**), the progression-free survival (PFS) (**H**), and the post-progression survival (PPS) (**I**) curves of FBXO2 were plotted for all OV patients in the Kaplan–Meier Plotter database (http://kmplot.com/).

We previously used the online GEPIA website to detect the expression of F-box protein family members and found that FBXO2 was one of the top three highly expressed FBPs in OV tissues [6]. However, the expression and functions of FBXO2 in OV have not been explored. The Cancer Genome Atlas (TCGA) database showed that the mRNA level of FBXO2 was relatively elevated in OV tissues (Fig. 1A). The high expression of FBXO2 was further confirmed in two independent cohorts of OV patients (GSE66957; GSE69428) (Fig. 1B, C). Interestingly, we found that FBXO2 was selectively highly expressed in OV among most tumor types from TCGA database, although its low expression was more common in some other tumors, suggesting that FBXO2 may play a unique role in OV (Fig. 1D). In addition, the Encyclopedia of Cancer Cell Lines (CCLE) database shows that FBXO2 is selectively highly expressed in most OV cell lines (Fig. 1E). Moreover, we assessed FBXO2 protein levels in human tissues from 10 cases of OV patients by IHC staining and found that FBXO2 protein levels were also elevated in OV tissues in related to normal ovarian tissues (Fig. 1F). We next used the Kaplan–Meier Plotter database (http://kmplot.com/) to analyze the prognostic value of FBXO2 in OV patients. We found that OV patients with high FBXO2 expression levels were significantly associated with relatively poor OS (Fig. 1G), PFS (Fig. 1H), and PPS (Fig. 1I). Together, these results indicate that FBXO2 is upregulated in OV and high levels of FBXO2 are related to the poor prognosis of OV patients.

### SOX6 functions as a positive regulator of FBXO2

**Fig. 2:**
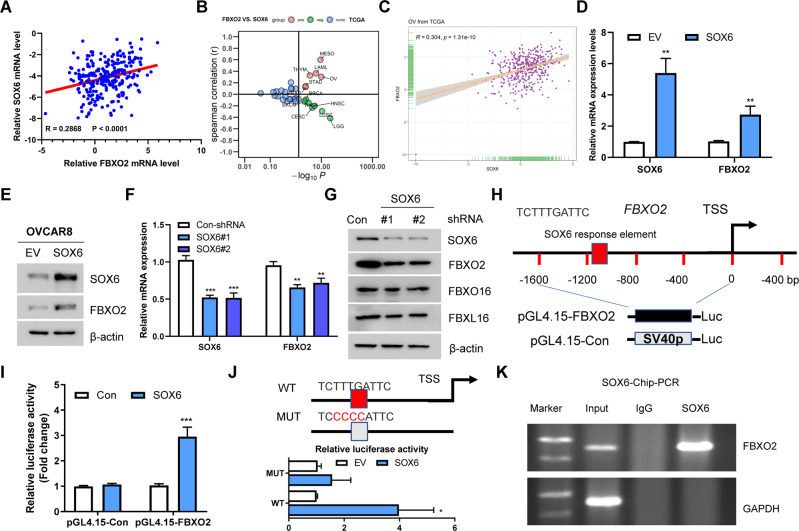
SOX6 functions as a positive regulator of FBXO2. **A** The mRNA expression correlation between FBXO2 and SOX6 in GSE32062 dataset. **B** The spearman correlation between the expression of FBXO2 and SOX6 in all tumor types in TCGA. Red represents positive expression and green represents negative expression. **C** The mRNA expression correlation between FBXO2 and SOX6 in OV from TCGA database. **D**, **E** The mRNA and protein levels of FBXO2 and SOX6 in A2780 cells transfected with indicated plasmids were determined by real-time PCR and immunoblotting, respectively. **F**, **G** The mRNA and protein levels of FBXO2 and SOX6 in A2780 cells transfected with Con-shRNA or SOX6-shRNA were determined by real-time PCR and immunoblotting, respectively. **H** Schematic diagram shows human FBXO2 gene promoter and one putative SOX6 binding site. TSS: transcription start site. **I** pGL4.15-Con or pGL4.15-FBXO2 plasmids were co-transfected with either FLAG-Con or FLAG-SOX6 into 293T cells for 36 h. The luciferase activity was then measured. **J** The human FBXO2 promoter contains one potential binding site for SOX6 was highlighted with RED. The mutant site was labeled with gray. FLAG-SOX6 was co-transfected with pGL4.15-Con or pGL4.15-FBXO2 plasmids into 293T cells for 36 h. The luciferase activity was then measured. **K** ChIP assay shows enrichment of SOX6 at the human FBXO2 promoter in A2780 cells.

To identify the upstream regulators of FBXO2 in OV, we first analyzed the co-expressed genes of FBXO2 in OV tissues (GSE32062) and found that SOX6 was the only transcription factor among the top 100 co-expressed genes (Fig. 2A). Next, we showed the expression of SOX6 and FBXO2 was also positively correlated in some tumor types in the TCGA database, including OV (Fig. 2B, C), suggesting that SOX6 may be involved in the regulation of FBXO2 expression in OV. Indeed, SOX6 overexpressing up-regulated both the mRNA and protein levels of FBXO2 (Fig. 2D, E), and silencing SOX6 generated the opposite effects (Fig. 2F, G). The core DNA sequence motif ATTGTT is mainly recognized by SOX transcription factors including SOX6 [10] and we found a putative SOX6 response element in the promoter region of FBXO2 (Fig. 2H), indicating that SOX6 may recognize it to regulate FBXO2 mRNA expression. Indeed, overexpression of SOX6 can induce 3–4 times the luciferase activity of the FBXO2 promoter (Fig. 2I). Moreover, the mutation of this putative motif largely prevented SOX6-driven FBXO2 promoter activity (Fig. 2J). Importantly, chromatin immunoprecipitation (ChIP) assay showed the occupation of SOX6 at the FBXO2 promoter where the putative SOX6 response elements located (Fig. 2K). Together, we conclude that SOX6 positively regulates FBXO2 expression in OV.

### FBXO2 is required for OV growth both in vitro and in vivo

**Fig. 3:**
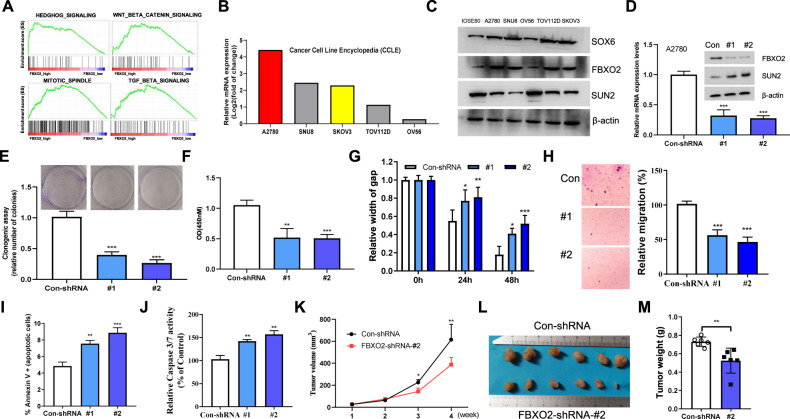
FBXO2 is required for OV growth both in vitro and in vivo. **A** Gene set enrichment analysis (GSEA) showed the enrichment of several signaling pathways in the FBXO2 high expressing OV tissues from TCGA OV database. **B** Relative FBXO2 mRNA expression in several OV cell lines obtained through the CCLE website. **C** The lysates of the indicated OV cells were immunoblotted with indicated antibodies. **D** The mRNA and protein levels of FBXO2 in A2780 cells stably expressing Con-shRNA or FBXO2-shRNA were determined by real-time PCR and immunoblotting, respectively. **E** Quantification of a colony-formation assay of the A280 cells from (**D**). **F** Cell proliferation in A2780 cells from (**D**) assessed by CCK-8 assay after 72 h. **G** The gap width in A2780 cells from (**D**) was measured at 24 h and 48 h, respectively. **H** The relative migration rate of A2780 cells from (**D**). **I** A2780 cells transfected with or without FBXO2-specific shRNAs were analyzed by FACS with Annexin V-PI assay. The graph represents the percentage of Annexin V positive cells. **J** Caspase3 and Caspase7 activity were measured in A2780 cells transfected with or without FBXO2-specific shRNAs. The *y*-axis indicates the caspase3 and caspase7 activity over cell number. The value given for the caspase activity in control-infected cells was set as 100. **K** The growth curves of xenograft tumors derived from subcutaneously implanted A2780 cells transduced with or without FBXO2-specific shRNA-#2. **L** Nude mice were sacrificed four weeks after transplantation. The tumors were excised, washed, and photographed. **M** Tumor weights were measured after mice were sacrificed.

We next investigated the biological functions of FBXO2 in OV. Gene Set Enrichment Analysis (GSEA) showed that a variety of cancer-promoting pathways including HEDGEHOG_ SIGNALING and WNT_BETA_CATENIN_ SIGNALING were significantly enriched in the FBXO2 high expression group from TCGA OV cancer database (Fig. 3A), suggesting a cancer-promoting role of FBXO2. Before we tested this possibility, we searched the CCLE database to identify OV cell lines overexpressing FBXO2. We found that A2780 cells contained the relatively high level of FBXO2 mRNA (Fig. 3B, C). The expression of FBXO2 was then silenced by shRNAs (Fig. 3D), and we found that cells with FBXO2 silencing showed reduced colony formation and proliferation abilities (Fig. 3E, F), as well as decreased migration and invasion abilities (Fig. 3G, H). Moreover, the apoptotic proportion of FBXO2 knockdown cells was noticeably increased (Fig. 3I), and the caspas3/7 activity was enhanced (Fig. 3J). In line with these in vitro results, knockdown of FBXO2 significantly reduced the volume, size, and tumor of the subcutaneous tumors (Fig. 3K–M). Moreover, the similar phenotypes could also be observed in SKOV3 cells with FBXO2 silencing (Supplementary Fig. 1A–F). Therefore, these results suggest that FBXO2 regulates the proliferation of ovarian cancer by controlling the apoptosis of OV cells.

### FBXO2 interacted with glycosylated SUN2 protein

**Fig. 4:**
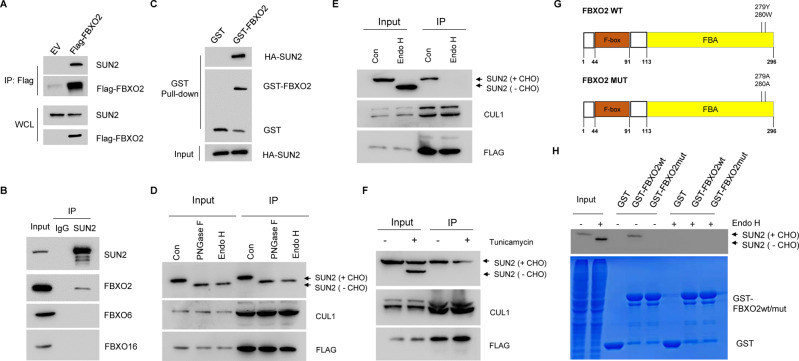
FBXO2 interacted with glycosylated SUN2 protein. **A** HEK293T cells were transfected with FLAG-Con or Flag-SUN2 for 36 h. MG132 was added 4 h before harvest. Cells were lysed and immunoprecipitated with Flag M2 beads. The beads-associated proteins were eluted by Flag peptide and subjected to immunoblot with anti-FLAG or anti-SUN2 antibodies, respectively. **B** The cell lysates of A2780 cells were subjected to immunoprecipitation with IgG or anti- SUN2 antibody and then detected by immunoblotting with indicated antibodies. **C** The HEK293T cells transfected with HA-SUN2 were lysed and then incubated with GST alone or GST-FBXO2 immobilized on GST-Sepharose beads. The bound protein was eluted with SDS loading buffer and detected by immunoblotting with indicated antibodies. **D** Lysates and immunocomplex from A2780 cells expressing FLAG-FBXO2 were treated with or without either Endo H or PNGase F for 1 h at 37 °C Samples were analyzed by immunoblotting with the indicated antibodies. CHO, carbohydrate oligosaccharide. **E** Lysates from A2780 cells expressing FLAG-FBXO2 were treated with (+) or without (−) Endo H at 37 °C for 1 h, and then were immunoprecipitated with anti-FLAG antibody and detected by immunoblotting with indicated antibodies. **F** A2780 cells expressing FLAG-FBXO2 were treated with (+) or without (−) 10 μg/mL tunicamycin for 36 h, lysates were immunoprecipitated with anti-FLAG antibody and detected by immunoblotting with indicated antibodies. **G** The diagram of FBXO2 WT and FBXO2 MUT proteins. Brown box indicates the F-box domain, yellow box indicates the FBA domain. **H** The HEK293T cells transfected with HA-SUN2 were treated with (+) or without (−) 10 μg/mL tunicamycin for 36 h. The cell lysates were incubated with GST alone or GST-FBXO2 (WT or Mut) immobilized on GST-Sepharose beads. The bound proteins were eluted with SDS loading buffer and detected by immunoblotting with indicated antibodies.

Previously, we have identified FBXO2-bound glycoproteins in HEK293T cells [8]. Among those glycosylated proteins, we focused on SUN2 protein, as it plays a tumor suppressor role in a variety of tumor cells by promoting apoptosis [11]. Firstly, we used immunoprecipitation (IP) assay to verify the interaction between FBXO2 and SUN2. We found that SUN2 can be readily detected in the immunoprecipitates of FLAG-FBXO2 (Fig. 4A). Secondly, the interaction between endogenous SUN2 and FBXO2 protein, but not FBXO6 or FBXO16, was observed in OV cells with the proteosome inhibitor MG132 treatment (Fig. 4B). In addition, GST-FBXO2 protein, but not GST protein alone, captured HA-SUN2 protein produced in HEK293T cells (Fig. 4C). To test the dependence of glycosylation for the interaction between SUN2 and FBXO2, we utilized two glycosidases including endoglycosidase Endo H and amidase PNGase F [12]. Removing the glycans from SUN2 protein by endoglycosidases generated a faster shift form of SUN2 on electrophoresis in both lysate and FLAG-immunoprecipitates of 293T cells expressing Flag-FBXO2 (Fig. 4D), suggesting FBXO2 interacted with SUN2 containing N-glycosylation. Furthermore, the cell lysate was treated with or without Endo H and then subjected to FLAG-IP (Fig. 4E). We found that after Endo H treatment, FBXO2 lost the ability to recognize SUN2 protein (Fig. 4E), suggesting that FBXO2 can only recognize glycosylated SUN2 protein. Furthermore, we utilized the protein glycosylation inhibitor tunicamycin, which could generate both glycosylated and non-glycosylated SUN2 forms (Fig. 4F). Our result clearly showed that FBXO2 still only recognized the glycosylated SUN2 protein (Fig. 4F). FBXO2 recognizes glycosylated proteins via its FBA domain (Fig. 4G) [13, 14]. We used the cell lysate with or without glycosidases treatment together with these GST-fusion FBXO2 proteins to perform GST-pulldown experiments, and found that FBXO2 WT can only recognize SUN2 protein without glycosidase treatment, while FBXO2 Mut cannot recognize any form of SUN2 at all (Fig. 4H). Therefore, all these results together conclude that FBXO2 specifically binds to glycosylated SUN2 protein via its FBA domain.

### FBXO2 targets glycosylated SUN2 for ubiquitination and degradation

**Fig. 5:**
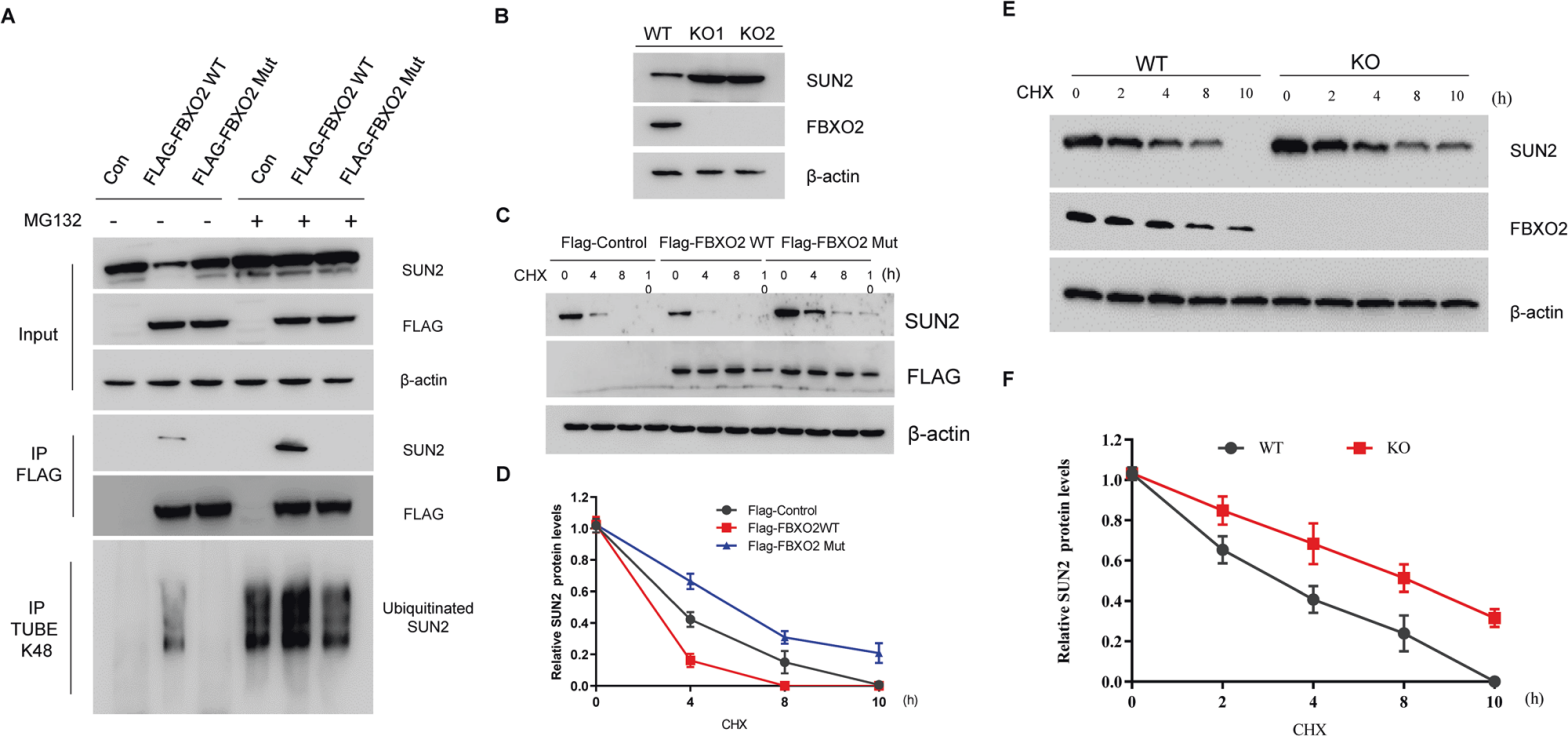
FBXO2 targets glycosylated SUN2 for ubiquitination and degradation. **A** HEK293T cells were transfected with indicated plasmids. MG132 was added 4 h before harvest where indicated. Lysates were immunoprecipitated with anti-FLAG antibody or by Tandem Ubiquitin Binding Entity (K48 TUBE HF) and immunoblotted as indicated. **B** Western blot analysis of A2780 FBXO2 WT (FBXO2^+/+^) cells or A2780 FBXO2 KO (FBXO2^−/−^) cells. **C** HEK293T cells were transfected with indicated plasmids and then treated with 20 μM CHX for the indicated time course. The cell lysates were examined by immunoblotting assay. **D** Statistic results of immunoblotting analysis from (**C**) were obtained by ImageJ software and were normalized to β-actin intensities, *n* = 3. **E** A2780 FBXO2 WT (FBXO2^+/+^) cells or A2780 FBXO2 KO (FBXO2^−/−^) cells were treated with Cycloheximide (CHX, 20 μM) for the indicated time course. The cell lysates were examined by immunoblotting assay. **F** Statistic results of immunoblotting analysis from (**E**).

To investigate whether SUN2 is regulated by FBXO2, FLAG-control, FLAG-FBXO2 WT, or FLAG-FBXO2 Mut plasmids were expressed into 293T cells with or without MG132 treatment. In control cells, we found that SUN2 protein was continuously degraded (Fig. 5A). Moreover, the interaction between FBXO2 and SUN2 protein suggested that FBXO2 might regulate the turnover of SUN2 protein. We found that overexpression of FBXO2 WT, but not FBXO2 Mut, resulted in a large loss of SUN2 protein (Fig. 5A). Interestingly, after administration of MG132, FBXO2 WT-induced SUN2 protein loss was prevented, and the interaction between FBXO2 WT and SUN2 was increased (Fig. 5A). However, FBXO2 Mut was unable to bind or regulate SUN2 protein with or without MG132 treatment. To further show the regulation of SUN2 by FBXO2, we generated FBXO2 knockout (KO) A2780 cells by CRISPR-CAS9 technology. As expected, FBXO2^−/−^ cells showed high and sustained levels of SUN2 compared to FBXO2 ^+/+^ A2780 cells (Fig. 5B). Thus, these results suggested that FBXO2 might be the E3 ligase for SUN2 degradation. Indeed, overexpression of FBXO2 WT, but not FBXO2 Mut, markedly reduced the protein half-life of SUN2 (Fig. 5C, D) and increase its ubiquitination modification (Fig. 5A, bottom panel). Moreover, compared with FBXO2 ^+/+^ A2780 cells, the half-life of SUN2 protein in FBXO2^−/−^ A2780 cells was significantly extended (Fig. 5E, F). Thus, these results indicate that FBXO2 negatively regulates the stability of SUN2 protein.

### FBXO2 promoted the progress of OV partially by degrading SUN2

**Fig. 6:**
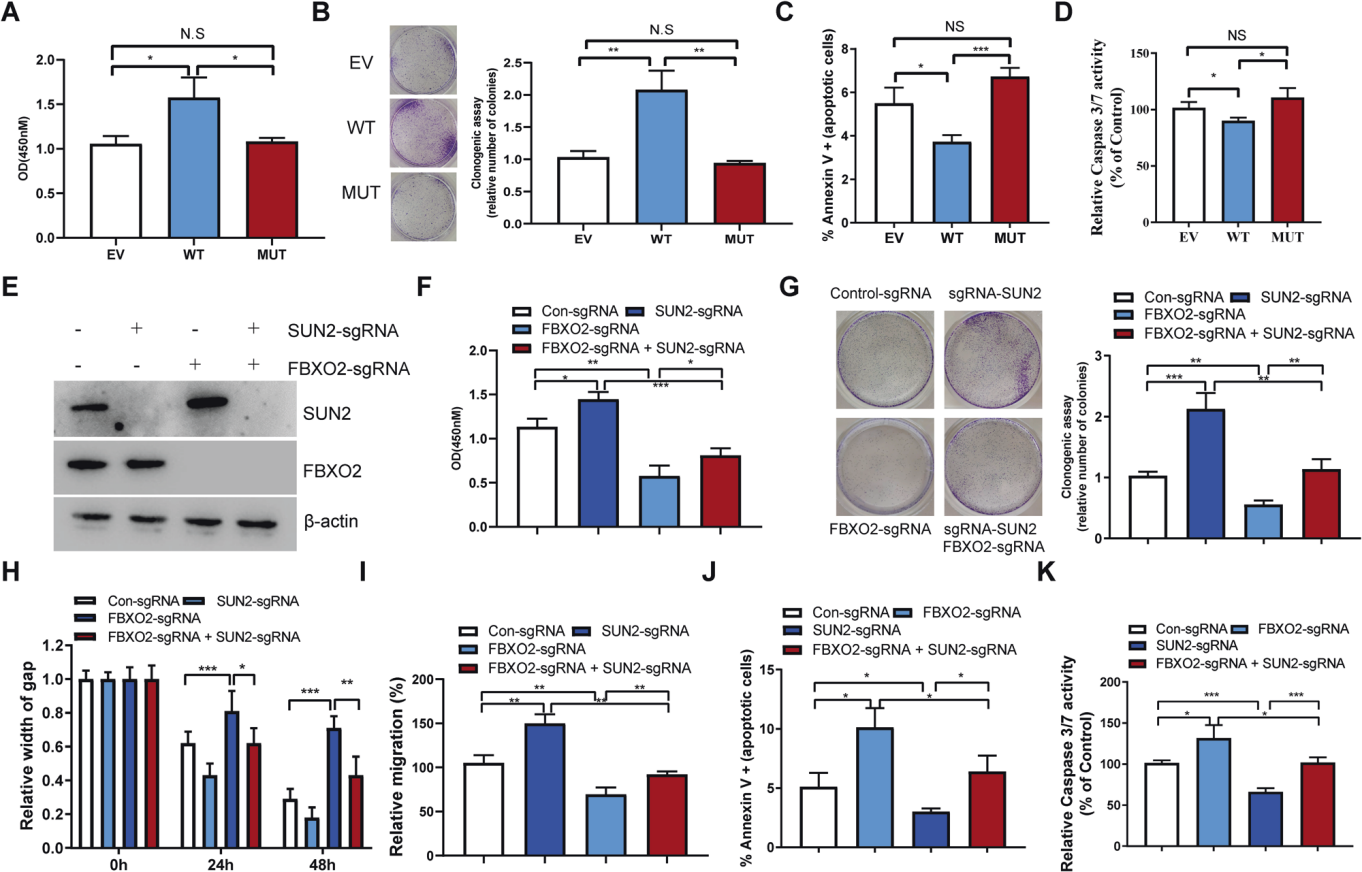
FBXO2 promoted the progress of OV partially by degrading SUN2. **A** Cell proliferation in A2780 cells transfected with EV or FBXO2WT or FBXO2 MUT plasmids were assessed by CCK-8 assay. **B** Quantification of a colony-formation assay of the A2780 cells in (**A**). **C** A2780 cells transfected with indicated plasmids were analyzed by FACS with Annexin V-PI assay. The graph represents the percentage of Annexin V positive cells. **D** Caspase3 and Caspase7 activity were measured in A2780 cells transfected with indicated plasmids. **E** The lysate of A2780 cells transfected with indicated sgRNAs was immunoblotted with the specified antibodies. **F** Cell proliferation in A2780 cells from (**E**) was assessed by CCK-8 assay. **G** Quantification of a colony-formation assay of the A2780 cells from (**E**). **H** The gap width in A2780 cells from (**E**) was measured at 24 and 48 h, respectively. **I** The relative migration rate of A280 cells from (**E**). **J** A2780 cells transfected with indicated sgRNAs were analyzed by FACS with Annexin V-PI assay. The graph represents the percentage of Annexin V positive cells. **K** Caspase3 and Caspase7 activity were measured in A2780 cells transfected with indicated sgRNAs.

**Fig. 7:**
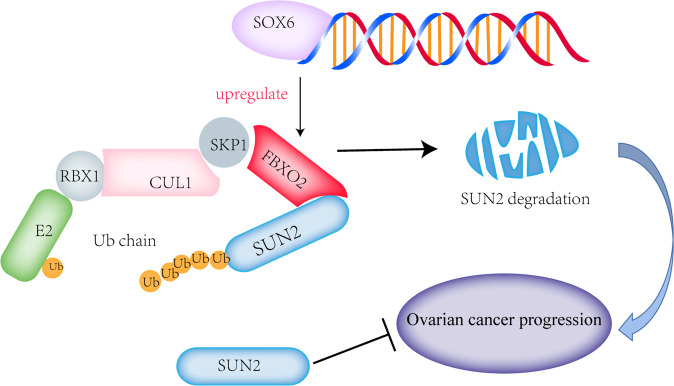
Working model. In this working model, we show that the transcription factor SOX6 promoted FBXO2 expression in OV. Abnormally highly expressed FBXO2 recognized and targeted glycosylated SUN2 protein for ubiquitination-depended degradation to promote the development of OV.

The loss of FBXO2 Mut’s ability to recognize and degrade SUN2 drove us to investigate whether the mutation of these two amino acids affects the biological function of FBXO2 in OV. We found that FBXO2 WT, but not FBXO2 Mut, significantly promoted cell proliferation and increased the numbers of the colonies (Fig. 6A, B). Moreover, stable expression of FBXO2 WT can reduce the proportion of apoptotic cells via decreased caspase 3/7 activity (Fig. 6C, D). However, FBXO2 Mut loses its ability to prevent OV cell apoptosis (Fig. 6C, D), suggesting that FBXO2 inhibits OV cell apoptosis via its FBA domain. We next investigated whether FBXO2 regulates OV progress through SUN2 degradation. We firstly assessed SUN2 protein levels in human tissues from 10 cases of OV patients by IHC staining and found that SUN2 protein levels were downregulated in OV tissues in related to normal ovarian tissues (Supplementary Fig. 2), suggesting SUN2 might play a opposite role of FBXO2 in OV. Next, we generated FBXO2 and SUN2 double KO cells via CRISPR-CAS9 technology (Fig. 6E). As expected, the cell growth defect phenotypes of FBXO2 KO cells were similar to that of FBXO2 silencing cells (Fig. 6F, G). Consistent with previously reports, knockout of SUN2 significantly promoted the proliferation of A2780 cells and increased the numbers of colonies (Fig. 6F, G) [15, 16]. Interestingly, knockout of SUN2 could also stimuli the proliferation of FBXO2 ^−/−^ A2780 cells (Fig. 6F, G). Depletion of FBXO2 gene significantly inhibited the migration and invasion ability of OV cells (Fig. 6H, I). However, the impaired migration and invasion abilities were largely restored when SUN2 was simultaneously depleted (Fig. 6H, I). Moreover, knockout of SUN2 largely decreased the activity of caspase 3/7 and prevented apoptosis of A2780 cells caused by FBXO2 depletion (Fig. 6J, K). In summary, these results indicate that the ability of FBXO2 to promote the proliferation of OV is at least partially dependent on the degradation of SUN2 (Fig. 7).

## Discussion

Previously, using protein purification approaches combined with mass spectrometry [8, 12], we found a series of glycosylation that specifically bound to FBXO2 or FBXO6, respectively. Although we observed that many glycoproteins could be recognized by both FBXO2 and FBXO6, SUN2 was not included, suggesting that FBXO2 may specifically recognize SUN2 proteins. SUN2 is a glycosylated protein and a key component of the junction of the nuclear skeleton and cytoskeleton (LINC) complex [17]. The defect of the LINC complex reduced the rigidity of the nucleus and cells and contributed to tumorigenesis [18]. Due to the relatively high expression of FBXO2 in OV tissues and its ability to specifically recognize the pro-apoptotic protein SUN2, we, therefore, focused on how FBXO2 regulates SUN2 protein and the biological significance of this regulation. Through a variety of biochemical methods, we showed that SUN2 is a bona fide substrate of SCF-FBXO2. As FBXO2 can recognize and degrade or even stabilize different glycosylated proteins, we cannot rule out that other substrates could also be involved in the cancer-promoting function of FBXO2. Indeed, many reported F-box proteins, such as β-trcp1, SKP2, and FBXW7, have different substrate proteins in the context of different tumor models [19, 20].

In summary, we have revealed a tumor-promoting role of FBXO2 and a connection with SOX6 in OV. FBXO2 promotes OV progression by ubiquitinating and degrading SUN2 to inhibit apoptosis. Thus, our results support a critical role of a novel SOX6-FBXO2-SUN2 axis in the development of OV, and targeting this axis may be an effective therapeutic mean of treating OV.

## Supplementary information

supplementary figure legends

s1

s2

original data

checklist

author contribution

## Data Availability

All data generated or analyzed in this study are included in this paper and can be obtained from the corresponding author according to formal requirement.
