# Supplementary material for: FBXO2 targets glycosylated SUN2 for ubiquitination and degradation to promote ovarian cancer development
Source: Cell Death Dis. 2022 May 7;13(5):442. doi: 10.1038/s41419-022-04892-9 (PMC9079088; doi:10.1038/s41419-022-04892-9)
Supplement: Supplementary file 1 — supplementary figure legends [file 41419_2022_4892_MOESM1_ESM.docx]

**Supplementary Figure legends**

**Supplementary Figure 1 in related to Figure 3**

1. The mRNA levels of FBXO2 in SKOV3 cells stably expressing Con-shRNA or FBXO2-shRNA were determined by real-time PCR. *** p<0.001.
2. The gap width in SKOV3 cells from (A) was measured at 24 hours and 48 hours, respectively. **p < 0.01, ***p < 0.001.
3. The relative migration rate of SKOV3 cells from (A). ***p < 0.001.
4. SKOV3 cells transfected with or without FBXO2-specific shRNA were analyzed by FACS with Annexin V-PI assay. The graph represents the percentage of Annexin V positive cells. **p < 0.01.
5. Caspase3 and Caspase7 activity was measured in SKOV3 cells transfected with or without FBXO2-specific shRNA. The y axis indicates the caspase3 and caspase7 activity over cell number. The value given for the caspase activity in control-infected cells was set as 100. *p < 0.05.
6. The growth curves of xenograft tumors derived from subcutaneously implanted SKOV3 cells stably expressing Con-shRNA or FBXO2-shRNA. n=6 per group, *p<0.05, **p<0.01.

**Supplementary Figure 2 in related to Figure 6**

The representative IHC staining images and the expression scores of SUN2 in normal ovarian tissues and ovarian cancer tissues from 10 OV patients.
