# Supplementary figures and images for: FBXO2 targets glycosylated SUN2 for ubiquitination and degradation to promote ovarian cancer development
Source: Cell Death Dis. 2022 May 7;13(5):442. doi: 10.1038/s41419-022-04892-9 (PMC9079088; doi:10.1038/s41419-022-04892-9)

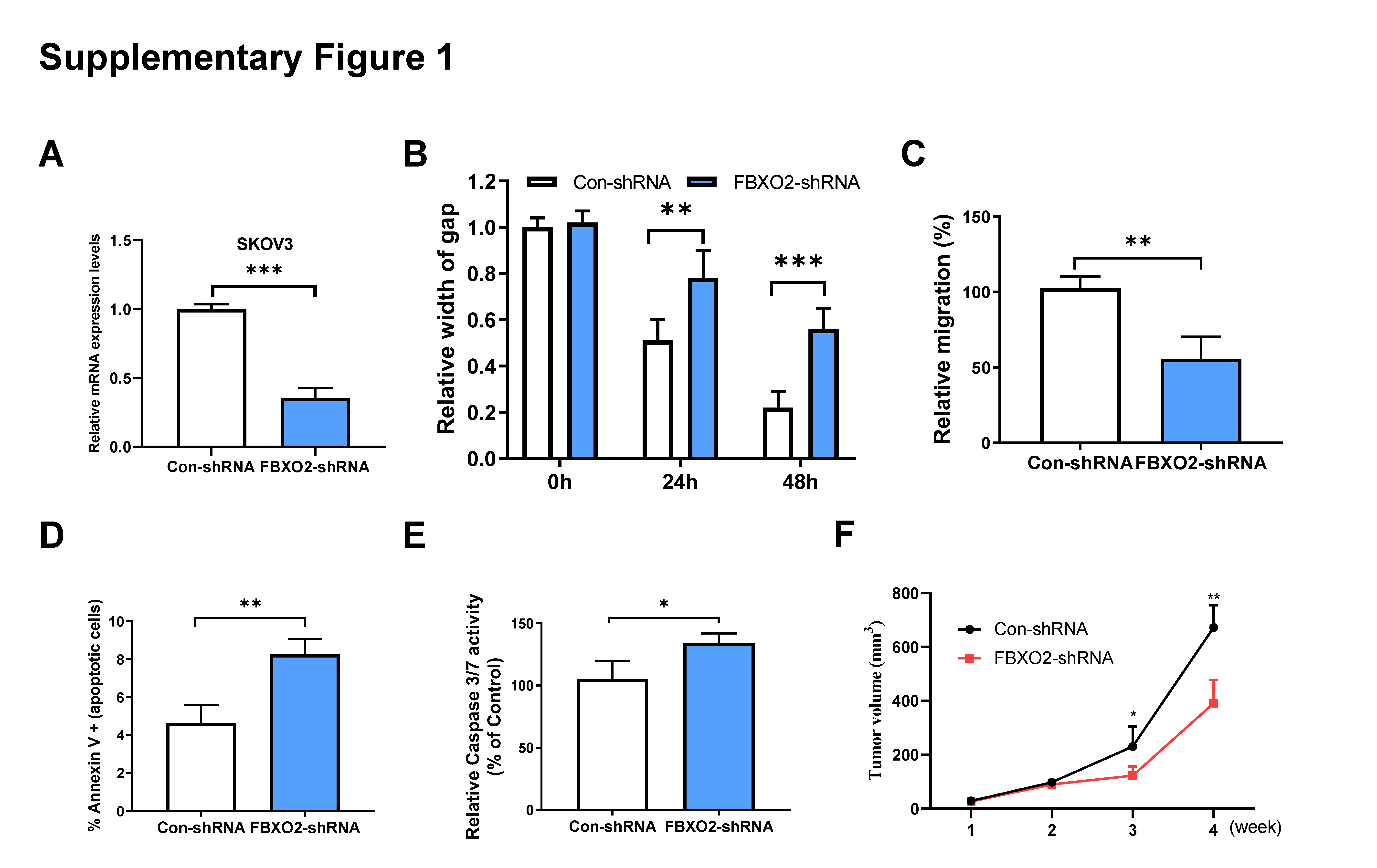

Supplement: Supplementary file 2 — s1 [file 41419_2022_4892_MOESM2_ESM.tif]

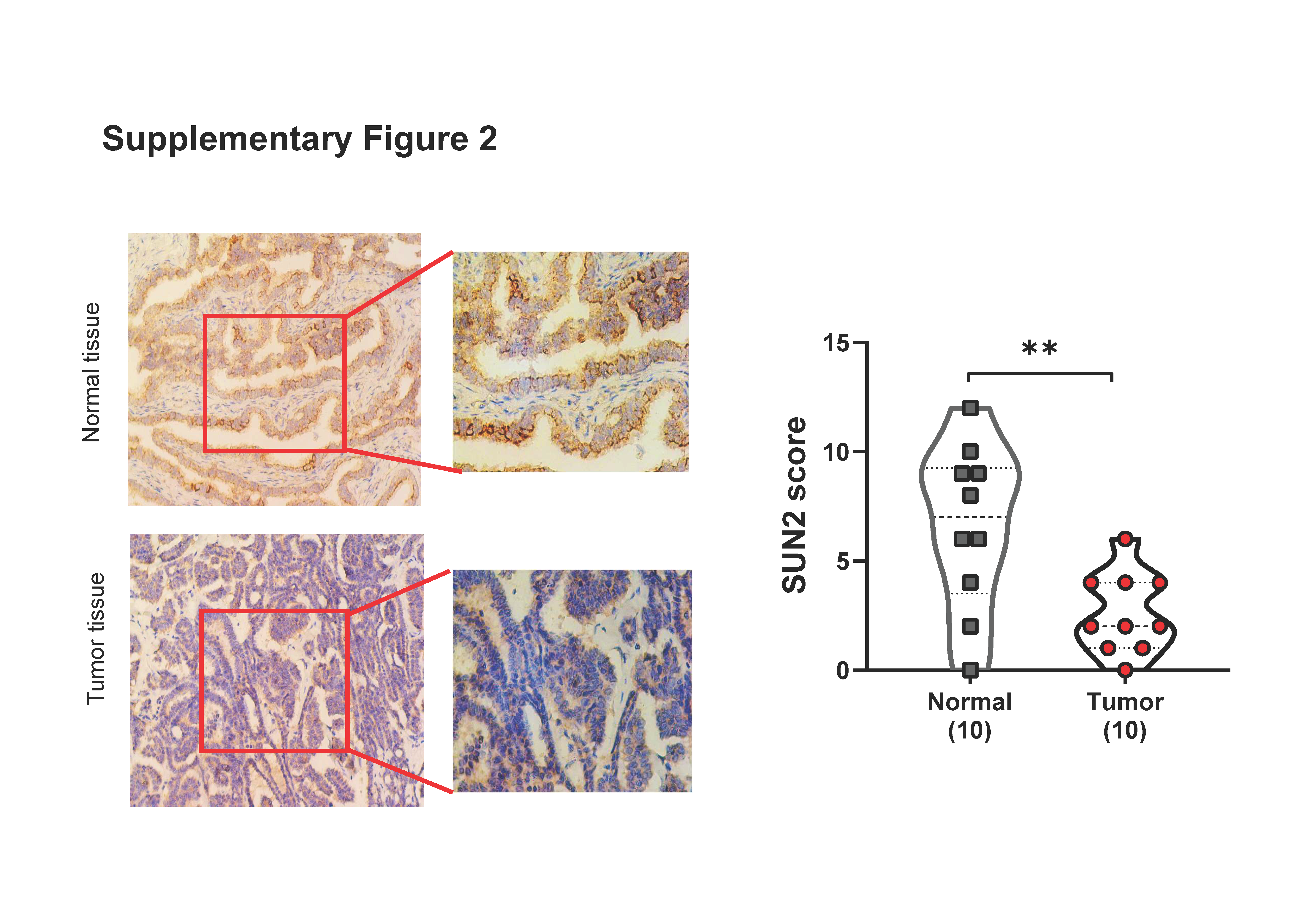

Supplement: Supplementary file 3 — s2 [file 41419_2022_4892_MOESM3_ESM.tif]

Figure 2

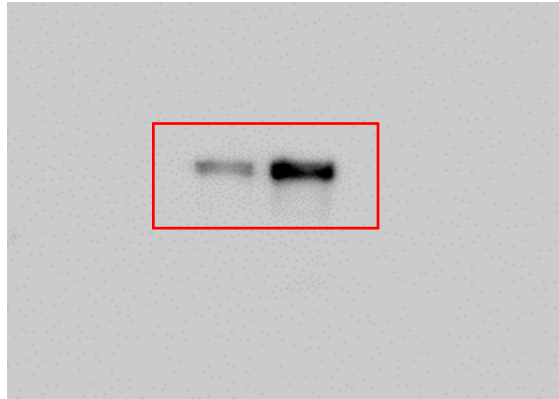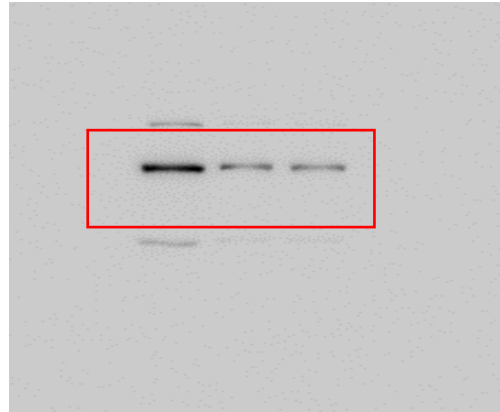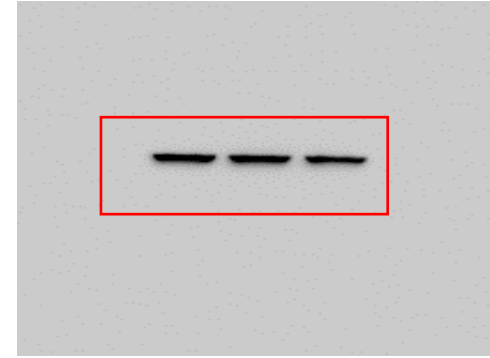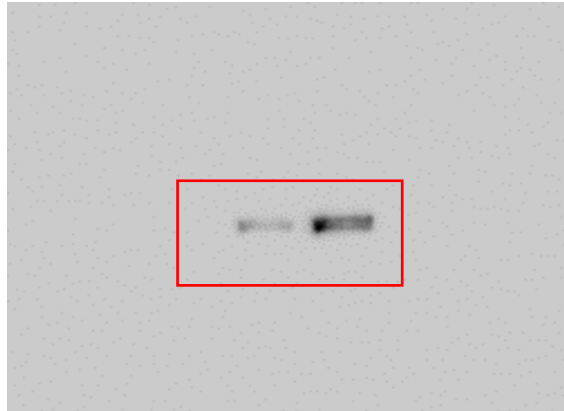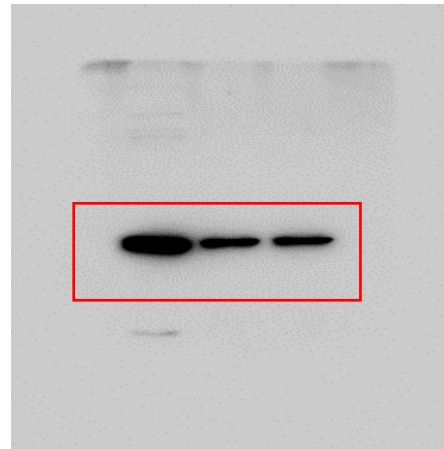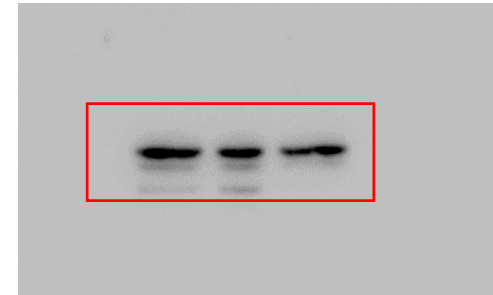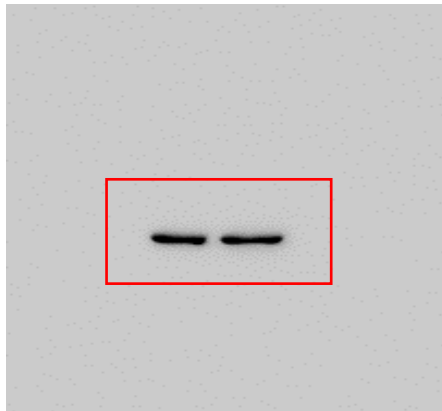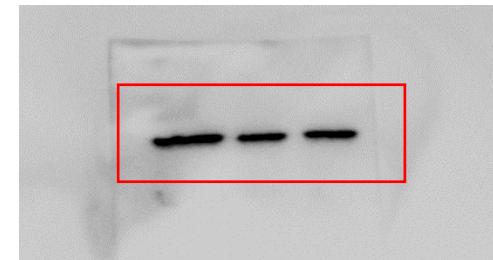

**Figure 3**

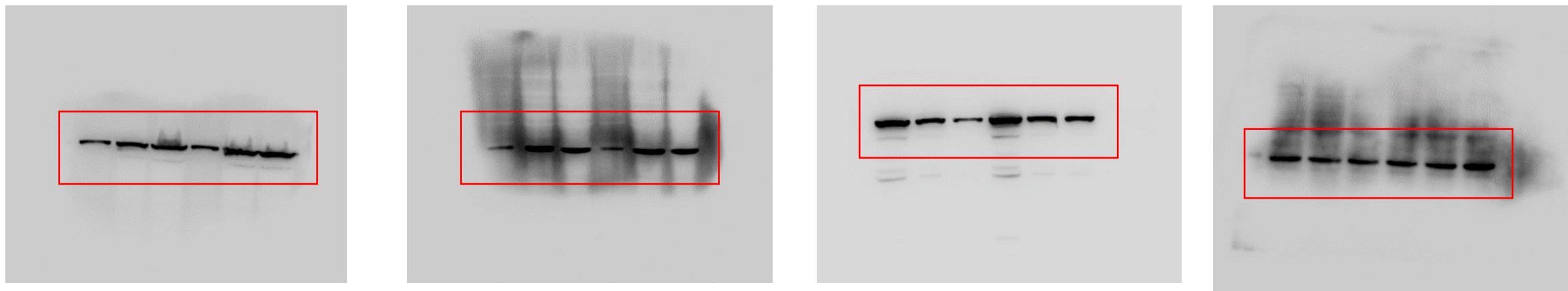

**Figure 6**

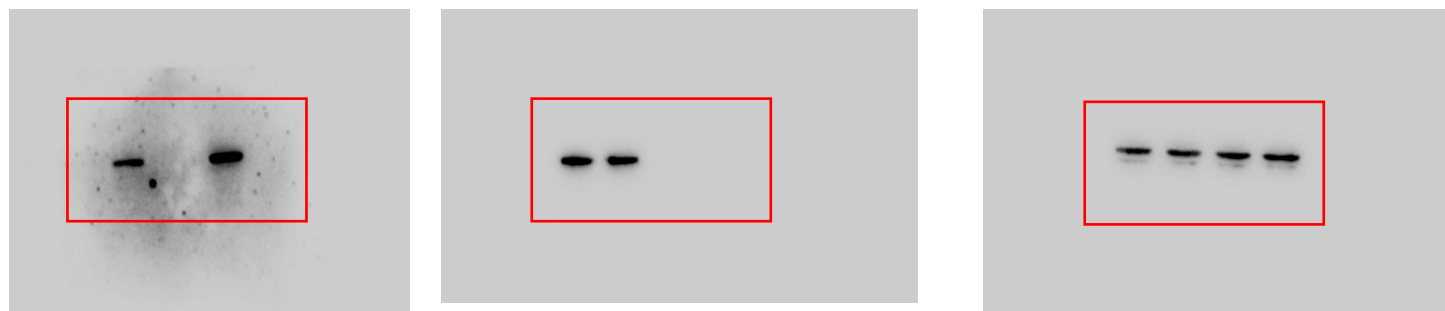

Figure 4

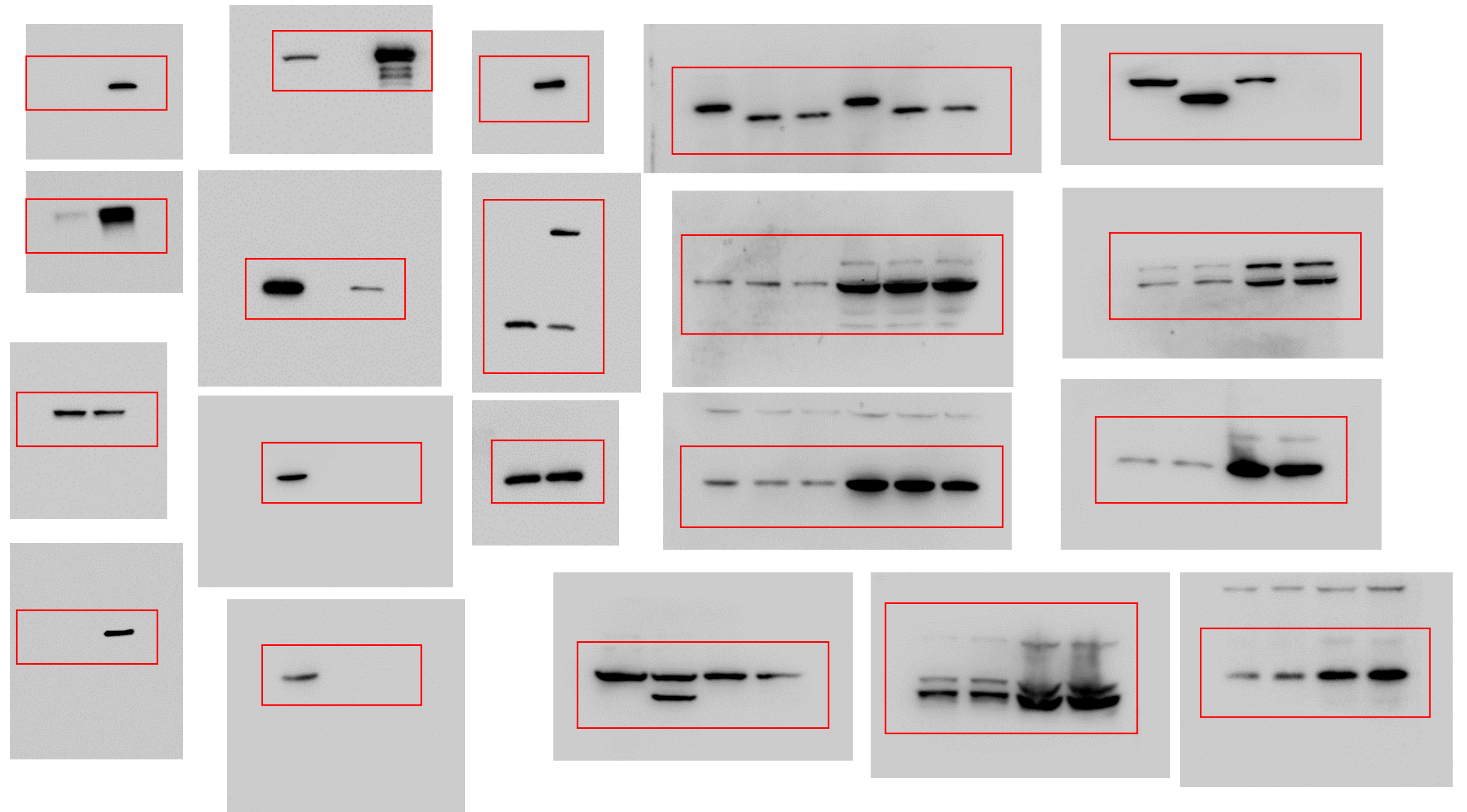

Figure 5

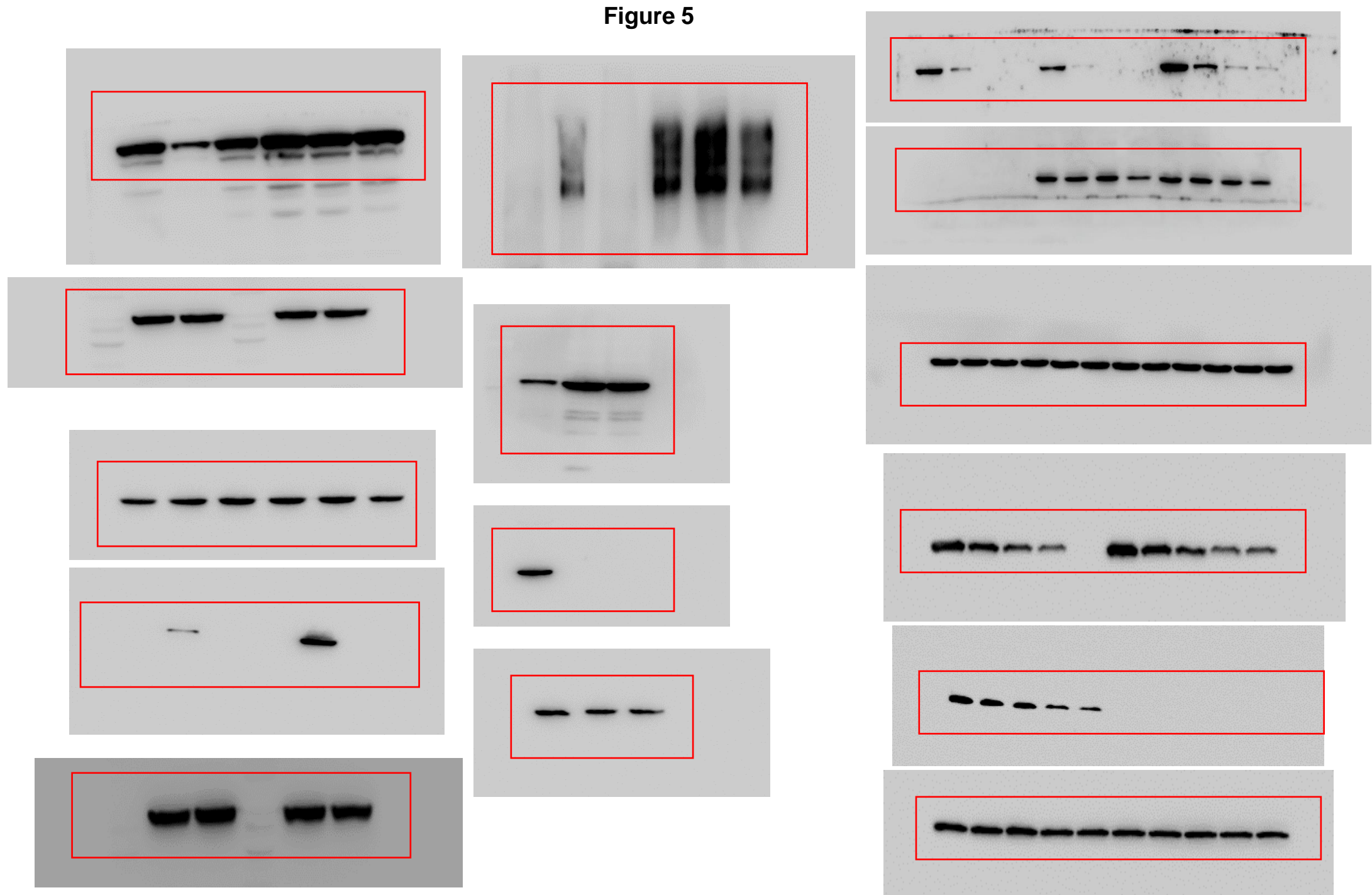

Supplement: Supplementary file 4 — original data [file 41419_2022_4892_MOESM4_ESM.pdf]
