# Supplementary material for: FBXO2 targets glycosylated SUN2 for ubiquitination and degradation to promote ovarian cancer development
Source: Cell Death Dis. 2022 May 7;13(5):442. doi: 10.1038/s41419-022-04892-9 (PMC9079088; doi:10.1038/s41419-022-04892-9)
Supplement: Supplementary file 6 — author contribution [file 41419_2022_4892_MOESM6_ESM.pdf]

DECLARATION OF CONTRIBUTIONS TO ARTICLE

Manuscript Number: CDDIS-21-3777R

Journal Name: *Cell Death & Disease* (the 'Journal')

Proposed Title of the Contribution:

FBXO2 targets glycosylated SUN2 for ubiquitination and degradation to promote ovarian cancer development (the 'Contribution)

Author(s):

Jing Ji, Jing Shen, Yuxin Xu, Mengru Xie, Qilan Qian, Teng Qiu, Wen Shi, Dexu Ren, Jinming Ma, Wei Liu, Bin Liu (the 'Authors')

For all *CDDis* articles, each person named as an author in the published version must be able to show he or she has contributed substantially to the article.

Authorship credit should be based on 1) substantial contributions to conception and design, acquisition of data, or analysis and interpretation of data; 2) drafting the article or revising it critically for important intellectual content; and 3) final approval of the version to be published. Authors should meet conditions 1, 2 and 3.

Any person who cannot be shown to have made a substantial contribution to the article cannot be listed as an author in the final version. The name of any person who is deemed to have made a minor contribution can, however, appear in the Acknowledgments section of the article.

Please complete the table below to indicate the contributions of all named authors to the manuscript.

Author Full Name:	Specification of Contribution to the Manuscript:
Jing Ji	performed most in vitro experiments and wrote the paper with Dr. Bin Liu
Jing Shen and Yuxin Xu	performed most in vivo experiments and IHC staning
Mengru Xie and Qilan Qian	performed some in vitro experiments and drew the working model
Teng Qiu and Wen Shi	performed protein purification and protein-protein interaction experiments,
Dexu Ren	performed online database searching experiments
Teng Qiu	performed some vector construction experiments
Jinming Ma	analyzed some in vitro data
Wei Liu	analyzed and discussed the data
Bin Liu	designed the whole study and wrote the paper

Please complete the table below to indicate the contributions of all named authors to the figures.

Figure 1:

Jing Ji, Jing Shen, and Dexu Ren generated the data and labelled the image, Bin Liu assembled the figure.

Figure 2:

Jing Ji, Yuxin Xu and Mengru Xie generated the data and labelled the image,Bin Liu assembled the figure.

Figure 3:

Jing Ji, Qilan Qian and Teng Qiu generated the data and labelled the image, Biu Liu assembled the figure.

Figure 4:

Jing Ji, Dexu Ren and Jinming Ma generated the data and labelled the image, Biu Liu assembled the figure.

Figure 5:

Jing Ji, Jing Shen, Qilan Qian, Teng Qiu, Wen Shi and Dexu Ren generated the data and labelled the image, Wei Liu, Bin Liu assembled the figure.

Figure 6:

Jing Ji, Jing Shen, Qilan Qian, Teng Qiu, Wen Shi, generated the data and labelled the image, Wei Liu, Bin Liu assembled the figure.

Signed for and on behalf of the Author(s):	Print Name:	Date:
	Bin Liu	02092020
